# Case report: Short-term psychotherapy for alexithymia in a patient with generalized anxiety disorder

**DOI:** 10.3389/fpsyt.2024.1342398

**Published:** 2024-04-15

**Authors:** Yufei Wang, Jinya Cao, Jing Wei

**Affiliations:** ^1^ Department of Psychological Medicine, Peking Union Medical College Hospital, Chinese Academy of Medical Science and Peking Union Medical College, Beijing, China; ^2^ 4^+^4 Medical Doctor Program, Chinese Academy of Medical Sciences and Peking Union Medical College, Beijing, China

**Keywords:** generalized anxiety disorder, psychotherapy, alexithymia, anxiety, case report

## Abstract

Alexithymia is common among patients with generalized anxiety disorder (GAD) and may negatively affect the efficacy of treatment. This case report described a sole short-term psychotherapy focusing on alexithymia for a GAD patient. The intervention extends over 3 weekly 50-minute sessions and incorporates components of: (a) understanding the basic categories of emotions and the importance of processing them consciously and building one’s own vocabulary of emotions; (b) developing skills in identifying and labeling emotions and learning to register both positive and negative emotions in daily life; (c) observing and interpreting emotion-related body sensations and learning to get in touch with, be empathetic to, and take care of one’s own inner feelings in daily life. The Hamilton Rating Scale for Depression (HRSD), Hamilton Anxiety Rating Scale (HAMA), and Toronto Alexithymia Scale (TAS) were used to evaluate depression, anxiety, and alexithymia before and after the sessions. The results suggested that the treatment was not only effective in reducing alexithymia helping the patient to clarify, identify and describe her feelings, but also effective in reducing anxiety and depression.

## 1 Introduction

Alexithymia is a set of cognitive and emotional features observed in patients with psychosomatic disorders, characterized by difficulties in perceiving, identifying, describing, and interpreting emotions in oneself and others (1, 2). This condition encompasses four main dimensions: (a) difficulty identifying and describing feelings; (b) difficulty distinguishing feelings from bodily sensations; (c) diminution of fantasy; and (d) concrete and minimally introspective (3). Individuals with alexithymia face difficulties in self-regulation and managing emotions due to diminished emotional awareness (4). Alexithymia have been implicated across a range of psychological disorders, with Leweke and colleagues finding a total prevalence rate of 21.36% in a mixed sample of psychiatric outpatients (5).

In the context of Generalized Anxiety Disorder (GAD), alexithymic traits are notably common, with prevalence rates varying between 12.5% and 58% among various subtypes of anxiety disorders (6). Patients with GAD and alexithymia often experience pronounced functional somatic symptoms and exhibit heightened somatosensory amplification and health-related anxieties (hypochondriasis), frequently presenting physical discomfort as their primary concern (7, 8). This is attributed to their difficulty in transitioning emotions from a somatosensory to a representational level, therefore using physical symptoms as an expression of psychological distress (9–11). Furthermore, these patients tend to have limited responses to pharmacotherapy (12, 13) and psychotherapy (14, 15). For limited response to pharmacotherapy, is partly due to the overlap in brain regions implicated in alexithymia and those targeted by selective serotonin reuptake inhibitors (SSRIs) (16–18). As for psychotherapy, is because these patients tend to have challenges in verbally articulating emotional experiences, affecting treatment motivation and efficacy (14, 15, 19).

Given the high prevalence of alexithymia among GAD patients and its detrimental impact on treatment outcomes, it is imperative to devise targeted interventions. Berking and colleagues advocate for a treatment model emphasizing emotional skills training, comprising nine specific objectives designed to enhance emotional awareness and regulation (20, 21). In alignment with this model, we have developed a concise, three-session psychological treatment plan for GAD patients with alexithymia, focusing on problem-solving and reducing dependency on long-term therapy. This case report details the application and effectiveness of this innovative approach, underscoring its potential in clinical practice.

## 2 Case information

### 2.1 Patient information

The patient was a 46-year-old heterosexual female, married with children, without any religious beliefs. She had no family history of mental illness, and no significant somatic medical conditions. The ethics committee of Peking Union Medical College Hospital has approved this study (Approval number: I-23PJ955), with assurance that data would be reported anonymously. A written informed consent has been obtained from the participant.

### 2.2 Chief complaint

The patient’s chief complaint was one year of somatic discomfort, and she was seeking treatment to alleviate her somatic symptoms.

### 2.3 History of present illness

The patient presented with symptoms of joint pain, dizziness, palpitations, and hot flashes one year ago, coinciding with the cessation of menstruation. Upon evaluation at the gynecology endocrinology outpatient clinic, a decreased estrogen level was detected, and the patient has been taking Fematone since then, which led to an improvement in joint pain but did not alleviate the other symptoms. A neurological consultation included a head magnetic resonance imaging (MRI) that revealed scattered punctuate ischemic lesions but no other abnormalities, and no specific treatment was needed. Over the past year, the symptoms have been variable, with a severe exacerbation one week ago. This exacerbation was characterized by a sensation of electrical sensations throughout the body, dizziness, head heaviness, palpitations, feelings of depression and anxiety, poor sleep quality with waking up around five in the morning, increased fatigue, and difficulty in concentrating on her daily office tasks. Laboratory blood tests, including complete hemogram, comprehensive metabolic panel, reproductive hormones and thyroid function, yielded results within the normal range. The patient also denied any substance use. There were no identifiable physical factors that could account for the patient’s symptoms.

### 2.4 Past medical history

The patient was diagnosed with multiple uterine fibroids with a maximum diameter of 1.2 cm during a medical examination two years ago. No specific treatment was administered. The patient denied a history of any other significant physical diseases. Additionally, there was no history of food or drug allergies.

### 2.5 Family members and personal growth history

The patient reported that the relationship between her parents in the original family was harmonious, and her parents were very focused on the patient’s upbringing. However, this focus was primarily on the patient’s academic performance. During childhood, the patient had aspirations to learn talents such as singing and dancing but felt that her academic achievements were only pursued due to her parents’ insistence. The patient’s parents believed that the sole focus should be on academics and did not fulfill the patient’s desires for other pursuits. The patient perceived herself as often feeling tense and unable to express herself freely.

In adulthood, the patient got married and had one child. Her overall relationship with her husband was also harmonious, but they had some disagreements regarding their child’s education. The patient believed that their child should be enrolled in extracurricular activities such as speech, recitation, and programming, while her husband did not share the same perspective on these matters.

## 3 Assessment

### 3.1 Psychological assessment

The patient was assessed using the Hamilton Anxiety Rating Scale (HAMA) (22, 23), the 17-item version of Hamilton Rating Scale for Depression (HRSD) (23, 24), and the 20-item version of Toronto Alexithymia Scale (TAS) (25, 26) as assessment tools. The patient scored 22 points on the HAMA scale, with 15 points in the psychic anxiety subscale and 7 points in the somatic anxiety subscale, indicating moderate anxiety. On the HRSD scale the patient scored 16 points, suggesting the presence of mild depression. As for the TAS scale, the patient scored 81 points, with sub-scale scores as follows: 17 points for difficulty identifying and describing feelings, 24 points for difficulty distinguishing feelings from bodily sensations, 24 points for diminution of fantasy, and 16 points for concrete and minimally introspective. The patient’s total score on the TAS-20 was significantly higher than the norm for Chinese females (66.94 ± 8.34) (26), indicating a notable presence of alexithymia.

### 3.2 Mental status exam

Appearance and Behavior: The patient maintained minimal eye contact, but responded coherently. Her facial expression reflected distress.

Mood and Affect: The patient exhibited a preoccupation with multiple concerns, leading to heightened anxiety and frequent startle reactions. She had difficulty relaxing and appeared restless. The patient was prone to jittery and sadness when influenced by external environment.

Thought Content: The patient described various somatic discomforts and occasionally reported a sense of loss of control. She expressed concerns related to somatic discomfort, life stressors, and associated worries.

### 3.3 Diagnostic assessment

Based on the criteria outlined in the fifth edition of diagnostic and statistical manual of mental disorders (DSM-5) for GAD (27), the diagnosis for this patient was generalized anxiety disorder.

### 3.4 Case conceptualization

**Figure 1:**
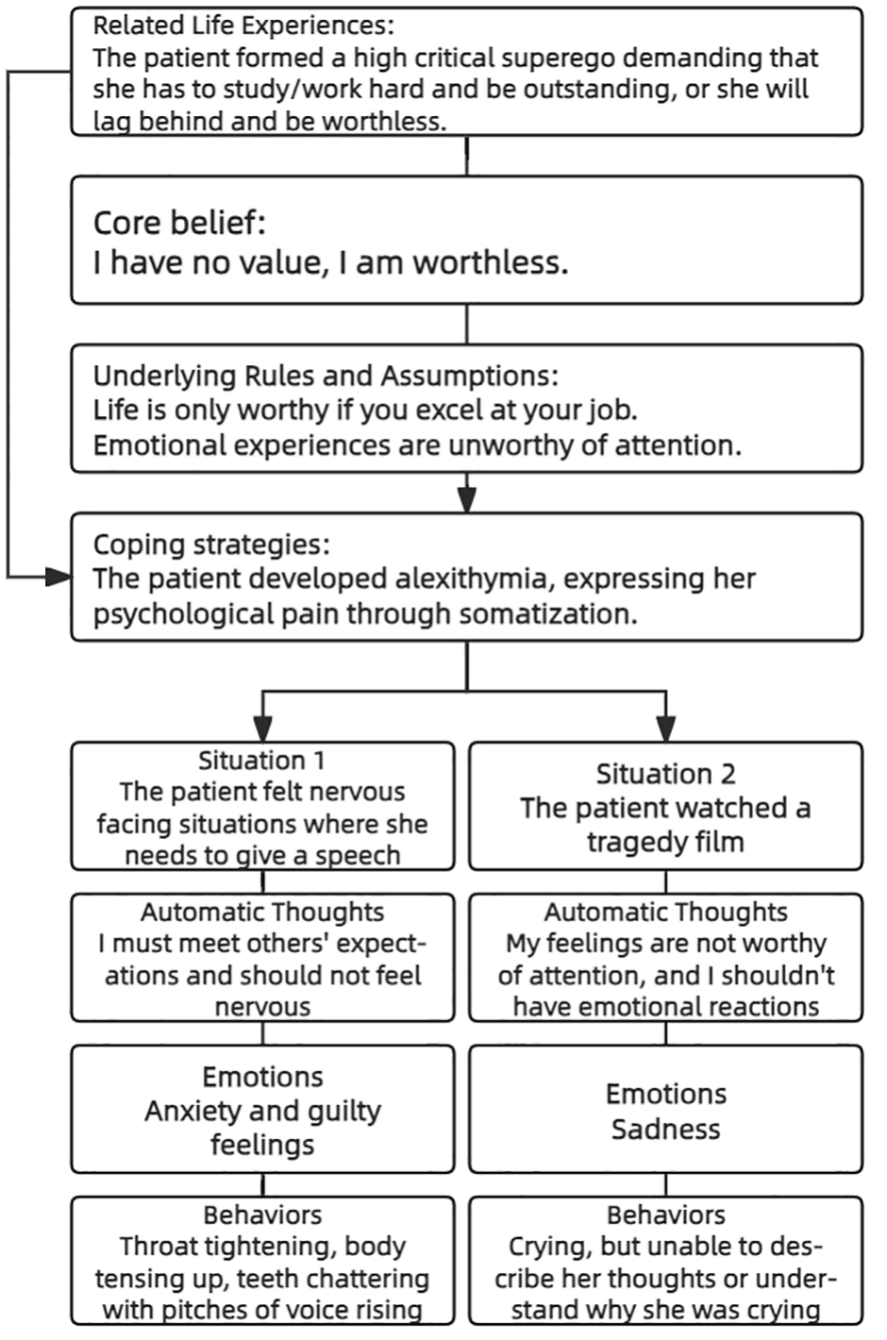
Cognitive conceptualization diagram.

Through her life experience, the patient formed a high critical superego demanding that she has to study/work hard and be outstanding, or she will lag behind and be worthless. She never learned to take care of her own emotional needs and perceives her own emotional experiences as unworthy of attention. Alexithymia made it difficult for her to articulate emotions and instead expressing psychological distress through somatization. The patient’s case conceptualization was illustrated in **Figure 1**.

## 4 Therapeutic intervention

### 4.1 Therapeutic goals and plan

The patient declined pharmaceutical recommendations. Based on the case conceptualization, a short term psychotherapy focusing on alexithymia was recommended: (a) to learn and respect her emotional feelings and get in touch with her own feelings; (b) to understand and narrate her somatic symptoms from an emotional perspective; and (c) to be empathetic to herself and thus build a more soothing part of superego.

A three-session treatment plan aligned with the aforementioned goals was specified as follows: (a) to understand the basic categories of emotions and the importance of processing them consciously and build her own vocabulary of emotions; (b) to develop skills in identifying and labeling emotions and learn to register both positive and negative emotions in daily life; (c) to observe and interpret emotion-related body sensations and learn to get in touch with, be empathetic to, and take care of one’s own inner feelings in daily life. The acting therapist (first correspondant author) is an associate professor in psychiatry holding a Doctor of Medicine degree, with a psychotherapeutic training in psychodynamic therapy and cognitive behavioral therapy (CBT). The co-designer of the study (second correspondant author) is a professor in psychiatry holding a Doctor of Medicine degree, with a psychotherapeutic training in Gestalt therapy. The therapists specialize in general hospital psychiatry, with a long time experience in treating common psychosomatic problems with integrative psychotherapeutic approaches.

### 4.2 Therapeutic sessions

Following discussions with the patient, a total of three therapy sessions were planned, each lasting 50 minutes. Homework was set in collaboration with the patient between sessions to facilitate a better post-therapy work.

#### 4.2.1 First therapy session

The initial session primarily focused on psychoeducation, encompassing two main areas: firstly, explaining to the patient the concept of emotions, their biological and psychological origins, their importance in our psychological function; secondly, the patient was then asked to name as many emotions as possible. The patient had great difficulty in abiding to the task of naming emotions. She very often switched to describe her physical symptoms and her concerns and ravels. The therapist attentively listened but did not give direct response to her worries, gently brought her back to the task, and facilitated the process by naming emotions in turn with the patient. Special attention was given to name not only negative emotions, but also positive emotions. At the end of the first session, homework was assigned to expand her vocabulary of emotions as far as possible through various ways such as reading short novels and discussing this topic with others.

#### 4.2.2 Second therapy session

The second session began by reviewing the homework completed after the previous session. This patient presents a very long list of carefully grouped emotions(just as a good student was supposed to present). The therapist encouraged and thanked the patient for her homework. The second session’s main task was to identify her own emotional experiences and label her emotions. With the help of her homework, we discussed which emotions could she identify from the last week’s work and life. The patient was asked to describe specific scenarios where these emotions were experienced and rated the intensity of emotions on a scale of 0-10. The patient listed various emotions, including feelings of grievance, disappointment, and relaxation. Special attention was given to her positive emotions to help her exemplify and register them. In the second session, the patient was more open to the task, but had difficulty in recalling finite emotions at the beginning. The therapist helped facilitate the process by describing own recent experiences. The patient gave a feedback that she felt being understood and in resonance with the therapist’s feelings and also felt relaxed at the end of this session. The homework for the second session was keep a diary of observing and logging emotions in daily life, with particular attention to positive emotions.

#### 4.2.3 Third therapy session

Building on the skills acquired in the first two sessions of naming and identifying emotions, the third session focused on guiding the patient to get in touch with bodily sensations and be empathetic to herself. The patient learned to observe, identify, and describe bodily sensations associated with emotions and to connect physical discomfort with emotional states. With the therapist’s guidance, the patient successfully described bodily sensations associated with the emotion of “nervousness,” including cold extremities, tight scalp, rapid breathing, muscle tension, and trembling voice and movements. The patient also connected her worries in the specific scenarios when these feelings occurred or worsened. She came to understand herself. The therapist demonstrated an attitude of being empathetic to these feelings and to her real life conditions and encouraged the patient try to sooth herself with her positive remembrances. The patient felt being accepted and decided to be more empathetic to herself in future. In the end, the therapist encouraged the patient to review the homework from previous sessions, summarize and apply the learned techniques in life and work.

#### 4.2.4 End and follow up

The patient reported that she improved a lot after the three sessions and agreed to feedback at one month and two months after the last session. The patient did not receive any other psychotherapy during or after the short term psychotherapy.

## 5 Therapeutic effectiveness assessment

### 5.1 Psychometric-based evaluation of therapeutic effectiveness

At one month after ending of therapy the patient gave a feedback that she kept doing fine. At two months after ending of therapy a reassessment with psychometric scales was done. The patient’s total score on HAMA decreased to 9, with psychic anxiety at 5 and somatic anxiety at 4; the total score on HDRS-17 decreased to 4; the total score on TAS-20 decreased to 76, with scores in the following dimensions: 14 points for difficulty identifying and describing feelings, 19 points for difficulty distinguishing feelings from bodily sensations, 16 points for diminution of fantasy, and 27 points for concrete and minimally introspective. Additionally, the patient reported a significant reduction in somatic symptoms and a marked improvement in sleep quality.

In summary, the patient’s anxiety and depressive symptoms has been largely relieved, and the severity of alexithymia was also reduced, with the TAS score now within the normal range (66.94 ± 8.34) (26) for Chinese women. Based on the above, the patient was assessed to be in a state of anxiety remission after treatment.

### 5.2 Patient perspective

**Figure 2:**
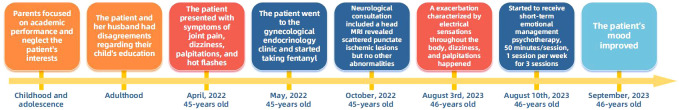
Timeline of case.

After treatment, the patient reported feeling emotionally much more stable, improved family relationships, and restored sleep and appetite, also enabling herself to engage in work. In terms of long-term goals, the patient has made positive changes cognitively, attitudinally, and behaviorally, acquiring the ability to recognize, describe and value her own emotions. The timeline of the case is shown in **Figure 2**.

## 6 Discussion

Alexithymia is common among patients with GAD (6) and may negatively affect the efficacy of both pharmacological and psychological treatments (12–15). This case primarily explored a short term psychotherapy designed focusing on alexithymia in in the treatment of a GAD patient. The outcomes tentatively indicate that this short-term psychotherapy approach could be beneficial in simultaneously reducing symptoms of anxiety and alexithymia in GAD patients.

Although alexithymia is considered to be associated with a wide spectrum of psychiatric disorders, it is particularly significant for mental disorders characterized by emotional components (5, 28, 29), such as GAD. Individuals with alexithymia exhibited heightened overall severity of disorder, a phenomenon particularly attributed to the increased severity of somatic symptoms associated with GAD (30). This correlation has been attributed to alexithymia originating from an inhibitory mechanism, which is believed to impede emotional processing within the right hemisphere, thereby diminishing the ability to verbalize emotional content within the left hemisphere (31, 32). Moreover, it has been established that GAD is specifically marked by deficiencies in emotional intelligence and in the experiences and regulation of emotions. This leads to an evasion of emotional stimuli processing, favoring the utilization of worry as a coping strategy in individuals with GAD (33). Consequently, alexithymic individuals with GAD may exhibit a diminished awareness of psychic anxiety, attributable to the hindrance of emotional processing and reduced capacity for verbalization of emotional content. Hence, the generalized anxiety in these subjects is predominantly manifested through pronounced somatic symptoms (30, 34). Our study provided treatment for a typical GAD patient exhibiting significant alexithymic characteristics and concurrent somatic symptoms, consistent with the aforementioned description. Improvements in anxiety and alexithymia were observed, while the mechanisms underlying these improvements warrant further investigation.

Whether alexithymia is modifiable remains a topic of ongoing controversy (35). One perspective suggests that alexithymia acts as a stable structure over time and often is independent of changes in somatic and psychological symptoms (36, 37). For example a large-scale, long-term Finnish study suggested that alexithymia exhibits high levels of both relative and absolute stability in adults, resembling a stable personality trait (38). Another view posits that alexithymia is substantially correlated with current anxiety and depression severity and can be changed through methods such as psychotherapy (39, 40). Norman and colleagues synthesized four studies intervening in alexithymia with mindfulness-based approaches through a meta-analysis and found that mindfulness intervention was an effective method to ameliorate alexithymia (41). The mechanisms by which psychotherapy ameliorates alexithymia are not yet fully clear. This may be because changes in alexithymia represent a secondary response to alterations in primary symptoms such as depression or anxiety. Alexithymia can be considered a defense or coping strategy against distressing emotions (42). Depression and anxiety might exacerbate features of alexithymia; however, these changes are reversible, and baseline alexithymia traits may remain unchanged after the alleviation of these disorders (38).

During the treatment of this case, we likewise observed a phenomenon that the patient experienced relief in anxiety and depression following interventions targeting alexithymia. This aligns with the current viewpoint that alexithymia may act as a mediator in a range of psychopathological phenomena including anxiety and depression (43–46). One hypothesis suggests that alexithymia may be a mediator between atypical interoception and anxiety (45). There is substantial research illustrating the correlation between interoception, alexithymia, and anxiety (47). Patients with alexithymia often exhibit deficits in interoception, such as inability to accurately report their own arousal states (48, 49) or to evaluate their feelings through subjective measures (50). Evidence from neuroimaging studies also indicated that individuals with alexithymia may exhibit atypical activity patterns in the interoceptive cortex (51). Palser and colleagues further discovered that alexithymia mediated the relationship between interoceptive sensibility and anxiety (45). They proposed that heightened sensibility to interoceptive signals, coupled with difficulty attributing these sensations to emotions, could lead to catastrophic interpretations of these sensations. Hence, alexithymia could become one of the intervention targets for anxiety symptoms in GAD patients. Nevertheless, given the predominance of cross-sectional studies in this area, the efficacy of alexithymia-targeted interventions for anxiety remains to be further validated through more rigorous research designs.

The findings presented in this study should be interpreted within the context of potential limitations. Firstly, as the study was a single-case report, the efficacy of this therapy should be approached with caution, necessitating further verification through future research based on Randomized Controlled Trial (RCT) designs. Secondly, the patient involved in the study was referred from somatic disease departments of a general hospital, which may not accurately represent the characteristics of GAD patients in psychiatric specialty hospitals. Subsequent RCT studies should employ a multicenter approach, recruiting both outpatient and inpatient GAD patients from psychiatric specialty and general hospitals to obtain a more representative sample. Lastly, since this study only reported the treatment process of a single GAD patient, it is challenging to establish the causal relationship between treatment, alleviation of alexithymia, and reduction of anxiety. Future RCT studies could consider employing a mediated cross-lagged design to further clarify the causal relationships among these variables.

In conclusion, we designed a three-session short-term psychotherapy integrating various elements of previous researches such as labeling emotions, correctly interpreting bodily sensations connected with emotions, being mindful of and empathetic to emotions. Besides, we also added the element of balanced attention to and registry of both negative and positive emotions in the therapy. The efficacy of this intervention deserves further test through RCT-based designs. The effectiveness of this intervention warrants further investigation through RCT-based studies.

## Data availability statement

The original contributions presented in the study are included in the article/supplementary material. Further inquiries can be directed to the corresponding authors.

## Ethics statement

The studies involving humans were approved by The ethics committee of Peking Union Medical College Hospital. The studies were conducted in accordance with the local legislation and institutional requirements. The participants provided their written informed consent to participate in this study. Written informed consent was obtained from the individual(s) for the publication of any potentially identifiable images or data included in this article.

## Author contributions

YW: Writing – original draft. JC: Conceptualization, Data curation, Funding acquisition, Methodology, Writing – review & editing. JW: Conceptualization, Funding acquisition, Project administration, Supervision, Writing – review & editing.
